# Sinus tarsi approach versus extensile lateral approach for displaced intra-articular calcaneal fracture: a meta-analysis of current evidence base

**DOI:** 10.1186/s13018-017-0545-8

**Published:** 2017-03-14

**Authors:** Hui Yao, Tangzhao Liang, Yichun Xu, Gang Hou, Lulu Lv, Junbin Zhang

**Affiliations:** Department of Orthopaedics, The Third Affiliated Hospital of Sun Yat-sen University, NO. 2693 Kaichuang Road, Guangzhou, 510100 People’s Republic of China

**Keywords:** Calcaneus, Fracture, STA, ELA

## Abstract

**Background:**

The extensile lateral approach (ELA) has been widely performed for displaced intra-articular calcaneal fractures (DIACFs), and wound complications remain a significant problem. As a minimal incision technique, the sinus tarsi approach (STA) was designed to overcome this disadvantage. There were already many reports about this approach but the conclusions were not completely consistent. Based on the current evidence, we performed this meta-analysis to compare the STA with ELA in the management of DIACF and expected to draw a certain and meaningful conclusion.

**Methods:**

All potentially relevant randomized controlled trials (RCTs) and cohort studies (CSs) were searched in the databases of PubMed, Embase, Cochrane Central Register of Controlled Trials (CENTRAL) and ClinicalTrial.gov. The desirable outcomes including wound complications, excellent and good rate, secondary surgery rate and Böhler’s angle were extracted. RCT studies were assessed using the Risk of Bias Tool recommended by the Cochrane Collaboration, and cohort studies were evaluated using the Newcastle–Ottawa Scale. The data of RCTs and cohorts were pooled respectively using the fixed-effect model or random-effect model. Mean differences with 95% confidence intervals (CIs) were calculated for continuous data, and relative risks (RRs) with 95% CIs were calculated for dichotomous data. Statistical heterogeneity was assessed with the Q test and *I* ^*2*^. Sensitivity analysis was developed to assess the reliability of pooled results.

**Results:**

Seven studies including two RCTs and five CSs were eligible for the meta-analysis. No matter RCTs or CSs, the pooled data all showed that STA group had a lower incidence of wound complications than that in the ELA group and no significant difference was found in excellent and good rate and the recovery of Böhler’s angle between the two groups. The CSs also showed that the STA group had a lower incidence of secondary surgeries than that in the ELA group.

**Conclusions:**

Through a STA, we not only can reduce the problems in wound healing but also achieve nearly the same adequate restoration of DIACF along with the similar functional outcomes compared with through an ELA.

## Background

Calcaneal fractures occur more often in young, active, persons performing manual labor while falling from a height and have a high socioeconomic impact. It is the most common fracture in the tarsal bones, accounting for 60% of all tarsal fractures [1] and approximately 2% of all fractures [2]. It has been reported that about 75% of calcaneal fractures are displaced intra-articular calcaneal fractures (DIACFs) [3].

Whether a DIACF should be managed surgically or not (conservatively) remains controversial. But a basic conclusion supported by certain randomized controlled trials (RCTs) [4, 5] and even many meta-analysis [6–8] has been drawn that surgical treatment can better reconstruct the anatomy of the calcaneus but bring a higher incidence of complications compared with the nonsurgical treatment. Surgical treatment can also lower the subtalar fusion rate and offer protection against early subtalar arthrodesis in DIACFs [7, 9].

The current reports provide strong support for the use of the ELA for the internal fixation operation of DIACFs [10–13], and it is even considered to be a standard treatment for DIACFs because the ELA provides excellent exposure of the fracture and allows direct reduction [14]. However, open reduction internal fixation (ORIF) through ELA comes with various complications among which the wound complications including edge necrosis, dehiscence, or deep infection should be paid particular attention. Many minimal invasive techniques were developed including percutaneous reduction internal fixation, external fixation and minimal incision techniques [15–19] to overcome this disadvantage. Percutaneous reduction internal fixation and external fixation were considered not able to accomplish and/or maintain the proper reconstruction of the fracture according to previous studies [15, 17, 20]. As a minimal incision technique, whether a sinus tarsi approach (STA) can aid to expose the fracture enough to achieve the adequate reconstruction of DIACF and meanwhile minimize the incidence of wound complications remains uncertain. Some RCTs [21, 22] and cohort studies (CSs) [23–27] were developed trying to answer this question, but the conclusions were not completely consistent. Based on the current evidence, we performed this meta-analysis to compare the STA with ELA for the management of DIACF and expected to draw a certain and meaningful conclusion for this question.

## Methods

This meta-analysis was conducted and reported in adherence to Preferred Reporting Items for Systematic Reviews and Meta-Analyses (PRISMA) [28].

### Study design and search methods

All published RCTs and CSs comparing STA with ELA for the management of DIACF were searched by two authors independently. PubMed, Embase, CENTRAL and ClinicalTrial.gov were searched for eligible reports. The search keywords were calcaneus, calcaneal, calcaneum, calcis, heel and hindfoot for study population; sinus tarsi, minimal, minimally, limited, mini, and small for test group; and extensile, extended, lateral, L-type, L-shaped, and conventional operation for control group. To find as many studies as possible, language, study design, publication status and date were not restricted in the search.

### Inclusion criteria

Studies meeting the following criteria were included: (1) Population: patients with DIACFs, closed, age older than 18 years, without previous calcaneal abnormalities or injuries (e.g. an infection or a tumour), co-existent foot injuries. (2) Interventions: reduction through a STA with the kind of fixation not cared. (3) Comparison: reduction through an ELA with the kind of fixation not cared. (4) Outcomes: studies that reported important postoperative outcomes, such as reduction quality, pain, function, or complications (at least one desirable outcome). (5) Study design: RCTs, prospective or retrospective CSs.

### Data extraction and quality assessment

The desirable outcomes including wound complications, excellent and good rate, secondary surgery rate and Böhler’s angle were extracted. The characteristics of the eligible studies including publication date, study location, study design, demographic data (sample size, average age and gender ratio), average follow-up time and surgical approach were also extracted. The risk of bias in included RCT studies was assessed using the Risk of Bias Tool recommended by the Cochrane Collaboration [29]. CSs were evaluated using the Newcastle–Ottawa Scale [30].

### Data synthesis and analysis

Data were analyzed separately for RCTs and CSs. The meta-analysis was performed using RevMan, version 5.3, software, and *p* < 0.05 was considered to indicate statistical significance. Mean differences with 95% confidence intervals (CIs) were calculated for continuous data, and RRs with 95% CIs were calculated for dichotomous data. Statistical heterogeneity was assessed with the Q test and *I* ^*2*^. Studies with an *I* ^*2*^ statistic of 25 to 50% were considered to have low heterogeneity, those with an *I* ^*2*^ statistic of 50 to 75% were considered to have moderate heterogeneity, and those with an *I* ^*2*^ statistic of >75% were considered to have a high degree of heterogeneity [31]. If *p* > 0.1 and *I* ^*2*^ < 50%, a fixed-effect model was used; otherwise, a random-effect model was used. Sensitivity analysis was developed to assess the reliability of pooled results. When the data extracted was not appropriate to pool, it was presented using a narrative analysis.

## Results

### Study selection process

**Fig. 1:**
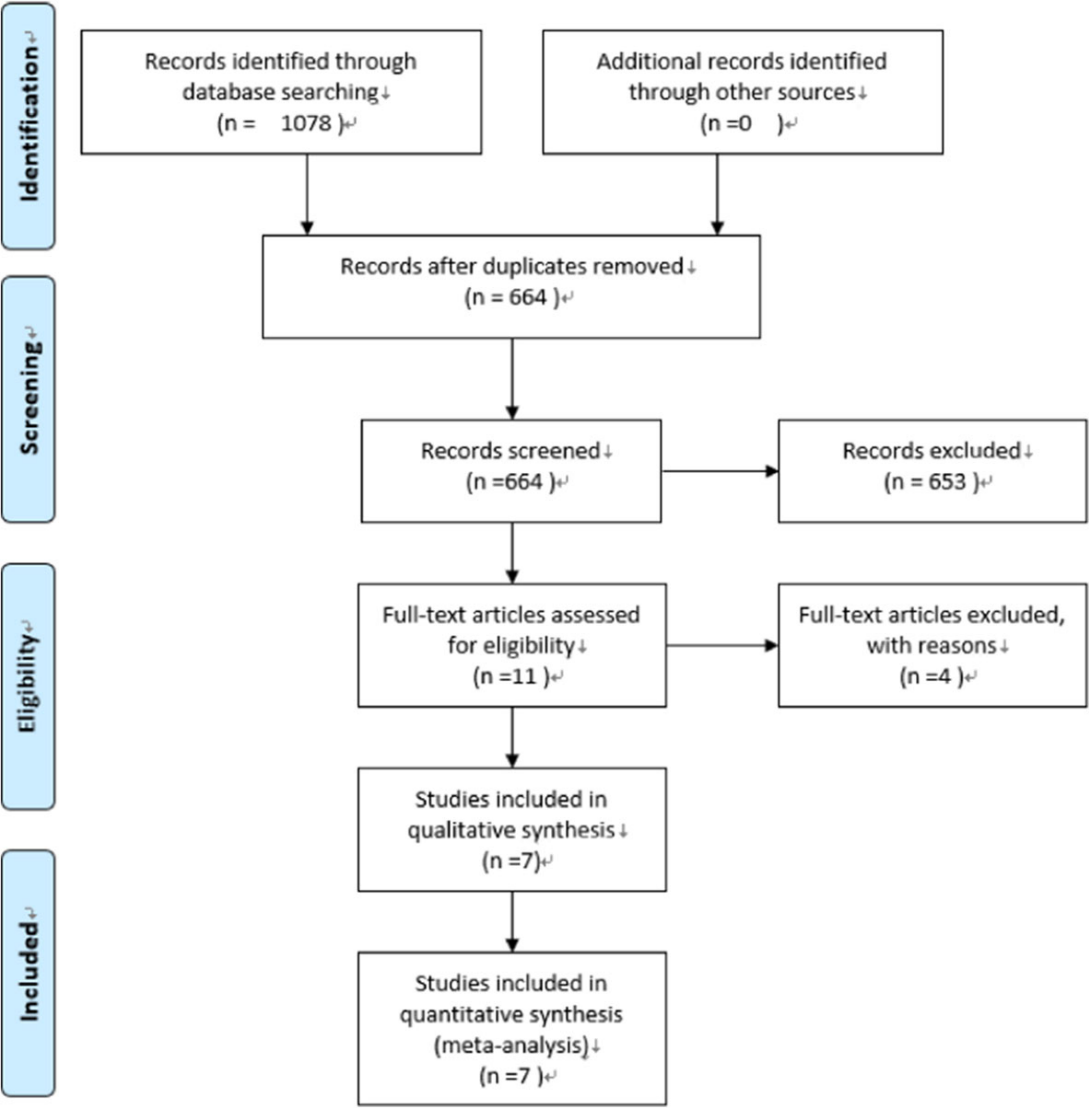
Flowchart of searches for studies (created using PRISMA 2009 Flow Diagram, version 2.1.3)

The process of study selection is presented in Fig. 1. A total of 1078 potentially relevant articles were identified, 467 from PubMed, 594 from Embase, 17 from CENTRAL and 0 from Clinicaltrial.gov. Of these, 664 records were left after removing the duplicates. After screening the titles and abstracts, 653 records were excluded leaving 11 reports which were retrieved in full text. Three records [32–34] were excluded because the study design was case control study, and 1 record [35] was excluded because of not using the STA. Seven studies including 2 RCTs [21, 22] and 5 CSs [23–27] were eligible for the meta-analysis.

### Study characteristics and quality assessment

The including studies were published between 2008 and 2016. A total of 784 patients were analyzed including 382 patients in the STA group and 402 patients in the ELA group. The mean durations of follow-up were all more than 12 months (Takasaka et al. [24] did not report the mean duration of follow-up, but their follow-up was at least 2 years).The total missing rate of follow-up data in RCT studies was 15.1% (26 of 172). Except the study of Wu et al. [23] whose sample size was very large and the missing data rate was up to 55.5% (410 out of 739), the other CSs all achieved the whole follow-up data without lost (Table 1). Risk of bias assessment of RCTs was presented in Table 2. Xia et al. [22] generated an adequately randomized sequence by coin tossing while Basile et al. [21] did not report how the randomized sequence was generated. When using the Newcastle–Ottawa Scale to assess the risk of bias of the cohort studies, the total scores were all higher than 5 indicating a low risk of bias (Table 3).

**Table 1:** Study characteristics

Study	Year	Location	Study design	Patients enrolled	Patients analyzed (STA/ELA)	Sex ratio (M/F)	Mean age (year) (STA/ELA)	Follow-up (month)	Missing data (%)
Basile et al. [21]	2016	Italy	RCT	45	38(18/20)	28/10	41.9/39.6	24/24	7(15.6%)
Xia et al. [22]	2014	China	RCT	127	108(59/49)	104/4	38/37	19/29	19(15.0%)
Wu et al. [23]	2012	China	CS	739	329(181/148)	307/22	39.4/41.5	12/12	410(55.5%)
Takasaka et al. [24]	2016	Brazil	CS	47	47(27/20)	NR	NR	NR	0(0%)
Kline et al. [25]	2013	USA	CS	112	112(33/79)	93/19	46.4/42.2	28/31	0(0%)
Weber et al. [26]	2008	Switzerland	CS	50	50(24/26)	NR	42.7/40	31/25	0(0%)
Yeo et al. [27]	2015	ROK	CS	100	100(40/60)	63/37	46/42	46/57	0(0%)

*M/F* male/female, *NR* not reported

**Table 2:** Risk of bias assessment of the RCTs

Study	Random sequence generation	Allocation concealment	Blinding of participants and personnel	Blinding of outcome assessment	Incomplete outcome data	Selective reporting	Other bias
Basile et al. [21]	Unclear risk	Unclear risk	Low risk	Low risk	Low risk	Low risk	Low risk
Xia et al. [22]	Low risk	Low risk	Unclear risk	Unclear risk	Low risk	Low risk	Low risk

**Table 3:** Risk of bias assessment of the CSs

Study	Selection					Outcome			
	Exposed cohort	Nonexposed cohort	Ascertainment of exposure	Outcome of interest	Comparability	Assessment of outcome	Length of follow-up	Adequacy of follow-up	Total score
Wu et al. [23]	*	*	*	*	**	*	*	*	9
Takasaka et al. [24]	*	*	*	*	–	*	*	*	7
Kline et al. [25]	*	*	*	*	**	*	–	–	7
Weber et al. [26]	*	*	*	*	**	*	*	*	9
Yeo et al. [27]	*	*	*	*	**	*	*	*	9

Risk of bias was assessed with use of the Newcastle–Ottawa Scale. "*" means a score of 1; "**" means a score of 2; the total score of this scale is 9. A higher overall score corresponds to a lower risk of bias; a total score of 5 or less indicates a high risk of bias

### Results obtained when only RCTs data were pooled

#### Wound complications

**Fig. 2:**
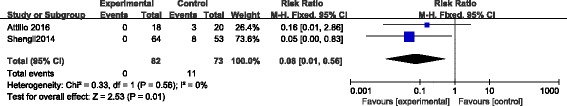
Forest plot of RR with 95% CIs for wound complications in RCTs

The incidence of wound complications was 0% (0 of 82) in the STA group versus 15.1% (11 of 73) in the ELA group (RR 0.08, 95% CI 0.01 to 0.56; *p* = 0.01, fixed-effect model), with no heterogeneity (*p* = 0.56, *I* ^*2*^ = 0%). The pooled data indicated that the incidence of wound complications in the STA group was significantly lower than that in the ELA group (Fig. 2).

#### Excellent and good rate

**Fig. 3:**
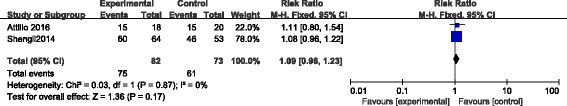
Forest plot of RR with 95% CIs for excellent and good rate in RCTs

The two RCTs all provided the data of excellent and good rate. Xia et al. [22] evaluated the final rate according to Maryland foot score while Basile et al. [21] using the American Orthopaedic Foot and Ankle Society (AOFAS) hindfoot score. Nevertheless, we thought the outcome was comparable. A total of 75 out of 82 fractures in the STA group compared with 61 out of 73 fractures in the ELA group were assessed excellent and good. However, no significant difference was found between the two groups (RR 1.09, 95% CI 0.96 to 1.23; *p* = 0.17, fixed-effect model), with no heterogeneity (*p* = 0.87, *I* ^*2*^ = 0%) (Fig. 3).

#### Böhler’s angle

**Fig. 4:**
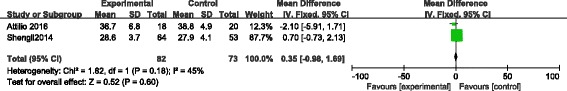
Forest plot of mean differences with 95% CIs for recovery of Böhler’s angle in RCTs

Two RCTs reported the postoperative recovery of Böhler’s angle. The pooled data showed that there was no significant difference between STA and ELA group (mean difference 0.35, 95% CI −0.98 to 1.69; *p* = 0.60, fixed-effect model), with no heterogeneity (*p* = 0.18, *I* ^*2*^ = 45%) (Fig. 4).

### Results obtained when only data of CSs were pooled

#### Wound complications

**Fig. 5:**
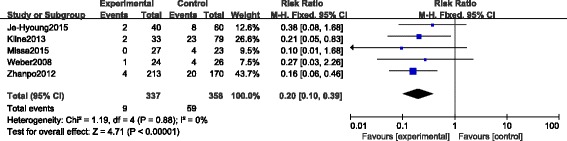
Forest plot of RR with 95% CIs for wound complications in CSs

All the CSs [23–27] reported the incidence of wound complications. The incidence of wound complications was 2.7% (9 of 337) in the STA group versus 16.5% (59 of 358) in the ELA group (RR 0.20, 95% CI 0.10 to 0.39; *p* < 0.00001, fixed-effect model), with no heterogeneity (*p* = 0.88, *I* ^*2*^ = 0%) indicating that the incidence of wound complications in the STA group was significantly lower than that in the ELA group (Fig. 5).

#### Excellent and good rate

**Fig. 6:**
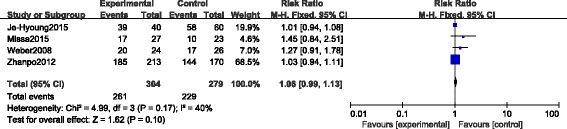
Forest plot of RR with 95% CIs for excellent and good rate in CSs

Four CSs [23, 24, 26, 27] provided the data of excellent and good rate, and all the investigators used the AOFAS score for the evaluation. A total of 261 of 304 fractures in the STA group compared with 229 of 279 fractures in the ELA group were assessed excellent and good. However, no significant difference was found between the two groups (RR 1.06, 95% CI 0.99 to 1.13; *p* = 0.10, fixed-effect model), with no heterogeneity (*p* = 0.17, *I* ^*2*^ = 40%) (Fig. 6).

#### Böhler’s angle

Four CSs [23–25, 27] reported the postoperative recovery of Böhler’s angle. Unfortunately, the data presented were not suitable to merge and we also failed to achieve the original data after contacting the main authors of these studies, but three of them [24, 25, 27] declared that they did not find any statistical significant difference between the STA and ELA group in the recovery of postoperative Böhler’s angle. Wu et al. [23] reported that the average postoperative Böhler’s angle was 28.51 (range 7–60) degrees in ELA group and 27.76 (range 9–43) degrees in STA group. The mean values were also very close.

#### Secondary surgeries

**Fig. 7:**
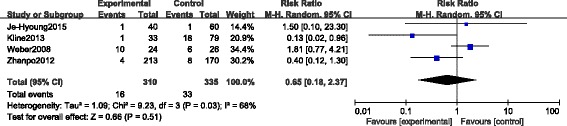
Forest plot of RR with 95% CIs for secondary surgeries in CSs

**Fig. 8:**
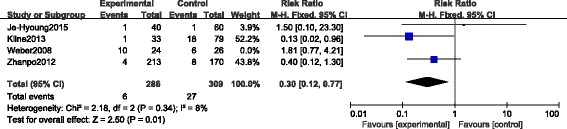
Forest plot of RR with 95% CIs for secondary surgeries in CSs excluding the study of Weber et al. [26]

There were four CSs [23, 25–27] which reported the secondary surgeries during the period of the whole follow-up. Yeo et al. [27] developed arthroscopic subtalar release for those complained of subtalar stiffness. The secondary surgeries reported by Kline et al. [25] included debridement, subtalar fusion for progressive painful arthritis and removal of symptomatic hardware. Wu et al. [23] removed the hardware for deep infection, wound edge necrosis and other severe defects. Weber et al. [26] reported the secondary surgeries including metal removal and subsequent subtalar arthrodesis. A total of 16 out of 310 fractures in the STA group compared with 33 out of 335 fractures in the ELA group underwent secondary surgeries. Because of detecting the heterogeneity with *p* = 0.03, *I* ^*2*^ = 68%, the random-effect model was performed and no significant difference was found between the two groups (RR 0.65, 95% CI 0.18 to 2.37; *p* = 0.51) (Fig. 7). To eliminate the heterogeneity and obtain a more objective result, we performed a sensitivity analysis by excluding the study of Weber et al. [26] whose incidence of secondary surgeries in the STA group (10 out of 24, 41.7%) was much higher than that in the ELA group (6 out of 26, 23.1%) correspondingly compared with Wu et al. [23] (4 of 213, 1.9% versus 8 of 170 4.7%), Kline et al. [25] (1 of 33, 3.0% versus 18 of 79 22.8%) and Yeo et al. [27] (1 of 40, 2.5% versus 1 of 60 1.7%). After excluding the study of Weber et al. [26], the heterogeneity disappeared (*p* = 0.34, *I* ^*2*^ = 8%), then we found the incidence of secondary surgeries in the STA group was significantly lower than that in the ELA group (RR 0.30, 95% CI 0.12 to 0.77; *p* = 0.01) using the fixed-effect model (Fig. 8).

## Discussion

According to the pooled data, the meta-analysis results of CSs were consistent with that of RCTs in wound complications, excellent and good rate and recovery of Böhler’s angle. The CSs also showed that the STA group had lower incidence of secondary surgeries than that in the ELA group. These findings indicates that through a STA, we not only can reduce the problems in wound healing but also achieve nearly the same adequate restoration of DIACF along with the similar functional outcomes compared with through an ELA.

Benefiting from excellent visualization and allowing access to manipulate and rigidly fix the fracture through direct reduction [36, 37], the ELA has been widely adopted in the treatment of DIACFs. However, this approach worsens the traumatic devascularisation of the central and anterior part of the lateral wall, as 45% of the calcaneal vascularity is derived from vessels entering at this site [38, 39]. Despite paying particular attention to delicate soft tissue management via the creation of full-thickness flaps and a “no touch” technique, the wound complications remain big troubles. Buckley et al. [36] showed a superficial wound complication of 17% and a deep infection rate of 5% in an operative group treated with open reduction and plate fixation through ELA. Howard et al. [40] reported an overall wound complication rate of 25% (57/226) on a retrospective review of 226 DIACFs. Folk et al. [41] reported 25% of wound complications in 190 patients with DIACFs. According to our meta-analysis results, the overall incidence of wound complications for the ELA group was 15.1% in RCTs and 16.5% in CSs which is consistent with the previous reports.

A STA is always made on a line from the tip of the lateral malleolus to the base of the fourth metatarsal with the length 3–5 cm. Advocators argued that it does not have the disadvantages of the ELA, as it lies in the internervous plane and respects soft tissue planes, leaving minimal space for a haematoma [26]. Holmes et al. [42] described using a STA and internal screw fixation to treat DIACF, with no problems with wound dehiscence, osteomyelitis, or surgical wound infection. Hospodar et al. [43] reported on 16 DIACFs treated with ORIF through the STA using screws for fixation. They detected no major wound complications. Kikuchi et al. [44] reported using the STA and a one-third tubular plate for 22 DIACFs. Their rate of superficial infection was 13.6% (3 of 22), no deep infections developed. The pooled data of this meta-analysis showed that the overall wound complication rate for the STA group was 0% in RCTs and 2.7% in CSs, which was also similar with the reported data.

This meta-analysis already proved that surgeries for DIACF through STA obviously reduce the risk of wound complications compared with through ELA, but whether a small incision about 3–5 cm long as the STA is enough for visualization to achieve adequate restoration of the DIACF may arise as a new question. This could be a serious problem because evidence from published data supports the concept that anatomic reduction and stable fixation of DIACFs will lead to the best possible outcomes [21].

For this question, many researchers presented their answers. Basile et al. [21] pointed out that the STA enables direct reconstruction of the posterior facet and anterior process, percutaneous reduction of the posterior tuberosity and strong fixation of the fracture with plate and screws despite limited exposure. Their results also showed no statistically significant differences in the quality of reduction (posterior facet and calcaneocuboid joint congruency, posterior tuberosity alignment) between the two groups. Wu et al. [23] described that a small lateral incision through the sinus tarsal permits a direct visualization of the articular surface, which allows an accurate reduction. Their recovery of Böhler’s angle was also similar between the two groups. Kline et al. [25] concluded that the minimally invasive STA for the reduction and fixation of DIACFs is safe and effective. No difference could be shown in their recovery of Böhler and Gissane angle between the two groups. Despite not providing the recovery of calcaneal angles, Weber et al. [26] described that the STA offers a window large enough to reduce and fix the joint and to control reduction of the tuberosity of the calcaneum. Loss of reduction of 2 mm or more of height, width or joint congruity or loss of more than 5° of Böhler’s angle did not occur in any patient in their groups. The data presented in our meta-analysis were also consistent with the abovementioned reports.

With the decrease of incidence of wound complications and equally adequate reconstruction of the fracture, equally good or better functional outcomes can be expected. Our meta-analysis results completely confirmed this supposition. Four cohorts [23, 24, 26, 27] in the present study all used the AOFAS score for the evaluation of excellent and good rate, and no significant difference was found between the two groups. Although the two RCTs adopted different evaluation methods for the functional outcomes, we thought the results were comparable only with regard to the excellent and good rate.

From the pooled data of cohort studies, we also found that the incidence of secondary surgeries in the STA group was significantly lower than that in the ELA group. First, we attribute the improvement to the lower incidence of wound complications. Fewer wound complications signify fewer follow-up therapies including secondary surgeries. Second, through a STA, we always implant fewer hardwares than through an ELA. Fewer hardwares may disturb the surrounding bone and soft tissue less. So, the rate of the symptomatic hardware removal and final subtalar arthrodesis for the traumatic arthritis is lower.

This meta-analysis has its own defects. First, the chief limitation of this study is that there are not enough RCT studies for the research. To increase the authenticity and reliability of the results, we included some high-quality CSs which would bring some methodological biases unavoidably. To minimize these biases, we pooled the data of RCTs and cohorts respectively, and the results were also analyzed respectively. Second, the different internal fixations may be the main confounding factor. Between the experimental group and control group, except the different surgical approaches, the hardware used for fixation is also different in some including studies. For example, some investigators used plate fixation in the ELA group but screw fixation in the STA group. Third, different researchers performed different evaluation methods and different durations of follow-up which may limit the accuracy of this study. So, more high-quality RCTs and high-level evidences are expected to give us more reliable guidance.

## Conclusions

Nevertheless, this study already drew a basic conclusion that through a STA, we not only can reduce the problems in wound healing but also achieve nearly the same adequate restoration of DIACF along with the similar functional outcomes compared with through an ELA.

## Abbreviations

AOFAS: American Orthopaedic Foot and Ankle Society
CENTRAL: Cochrane Central Register of Controlled Trials
CI: Confidence interval
CS: Cohort study
DIACF: Displaced intra-articular calcaneal fracture
ELA: Extensile lateral approach
NR: Not reported
ORIF: Open reduction internal fixation
PRISMA: Preferred reporting items for systematic reviews and meta-analyses
RCT: Randomized controlled trial
RR: Relative risk
STA: Sinus tarsi approach
